# Supplementary material for: Risk factors contributing to infection with SARS-CoV-2 are modulated by sex
Source: PLoS One. 2024 Feb 28;19(2):e0297901. doi: 10.1371/journal.pone.0297901 (PMC10901358; doi:10.1371/journal.pone.0297901)
Supplement: Attachment — Submitted filename: 2024-01-04_Response to the reviewers.docx [file pone.0297901.s002.docx]

**Response to the editor and the reviewers**

We thank the editor and both reviewers for their careful revision of our manuscript. We feel that the comments have helped to improve the text. Please find below responses to each of the comments by both reviewers.

As required by the editor, we have removed the figures from the main manuscript and we have uploaded the figures as separate files to the system.

Changes and corrections to the manuscript are indicated in blue in the manuscript. Apart from the corrections suggested by the reviewers, we have also corrected some of the numerical values of Table 2 that were erroneous, we have corrected the formula for the estimation of the sample size that was incorrect and we have added extra numerical details of the statistical analysis using MANOVA (page 13, lines 269-276), ANOVA (pages 14-15, lines 289-311) and Mann-Whitney U test (page 15, lines 313-326) in the Results section.

**Reviewer 1**

**- Avoid including citations in the abstract.**

*We have rewritten the abstract and excluded citations that were previously included. See lines 28-45 on pages 1-2 of the manuscript:*

Throughout the early stages of the COVID-19 pandemic in Mexico (August - December 2020), we closely followed a cohort of n=100 healthcare workers. These workers were initially seronegative for Immunoglobulin G (IgG) antibodies against SARS-CoV-2, the virus that causes COVID-19, and maintained close contact with patients afflicted by the disease. We explored the database of demographic, physiological and laboratory parameters of the cohort recorded at baseline to identify potential risk factors for infection with SARS-CoV-2 at a follow-up evaluation six months later. Given that susceptibility to infection may be a systemic rather than a local property, we hypothesized that a multivariate statistical analysis, such as MANOVA, may be an appropriate statistical approach. Our results indicate that susceptibility to infection with SARS-CoV-2 is modulated by sex. For men, different physiological states appear to exist that predispose to or protect against infection, whereas for women, we did not find evidence for divergent physiological states. Intriguingly, male participants who remained uninfected throughout the six-month observation period, had values for mean arterial pressure and waist-to-hip ratio that exceeded the normative reference range. We hypothesize that certain risk factors that worsen the outcome of COVID-19 disease, such as being overweight or having high blood pressure, may instead offer some protection against infection with SARS-CoV-2.

**- Consider whether this study may be considered salami publication, as its results could potentially have been integrated with those of Bueno-Hernández et al. (2022).**

*We have rewritten the introduction to summarize each of the 4 different study objectives and the corresponding results of the original publication. The present contribution focuses only on the 4th study objective of identifying risk factors in demographic, anthropometric, clinical, and physiological data for infection with SARS-CoV-2. The previous publication applied common univariate statistics to this data, the conclusions with respect to risk factors were counter intuitive and formed the research question for the present contribution. Here, we hypothesized that a more advanced and multivariate statistical approach would allow to reach a better understanding of our data. See lines 48-1126 on pages 2-5 of the manuscript:*

In a recent publication, we reported on results of a cohort of healthcare workers (HCW) from the Hospital General de México Dr. Eduardo Liceaga (HGMEL), who were exposed during the early phase of the COVID-19 pandemic in Mexico, from August to December 2020 [1]. The cohort underwent a baseline evaluation during which a variety of categorical and continuous variables were registered, including medical history, demographic, anthropomorphic, clinical and laboratory information, and working conditions. Possible infection with SARS-CoV-2 was detected at baseline, using an anti-SARS-CoV-2 Immunoglobulin G (IgG) antibody serum titers test and a quantitative polymerase chain reaction (qPCR) test. Possible infection was examined as well during the evaluation period in symptomatic participants using a qPCR test, and during a follow-up study six months later using an IgG test. The cohort included only participants who tested negative at baseline and was analyzed by groups defined post-hoc depending on the outcome of the IgG test at 6 months, using a group that remained seronegative (IgG-) and a group that became seropositive (IgG+). This previous publication had multiple study objectives. The first objective was to evaluate the incidence of SARS-CoV-2 infection in HCW of HGMEL, and to compare results from the qPCR and IgG tests. The incidence rate of SARS-CoV-2 infection was considerably higher in our cohort (58%) than in other healthcare populations as published by other authors (5-29%) [2-9]. Most reports used qPCR tests to detect positive cases. Since qPCR amplifies the genetic material of the virus, this methodology can only identify active infection cases, which may lead to underestimating asymptomatic patients and incidence rates among healthcare professionals. Therefore, one of our conclusions was that the incidence of SARS-CoV-2 infection in HCW should be assessed by IgG antibody measurements irrespective of having presented COVID-19 symptoms or not. The second objective was to analyze the incidence rate of infection in HCW depending on the workplace in the hospital. We found that HCW who were correctly wearing personal protection equipment (PPE), exhibited infection rates that were similar for high-exposure (e.g., emergency room or intensive care units) and moderate- and low-exposure areas (e.g., hospital administration or medical social work). In correspondence with previous studies [10,11], we concluded that the risk of SARS-CoV-2 infection in HCW is not necessarily associated with the workplace but rather with other factors such as the inaccurate use of PPE, and community exposure or contact with family members who are asymptomatic SARS-CoV-2 carriers. A third objective was to evaluate the prevalence of symptoms related to SARS-CoV-2 infection. We found that only 33% of IgG seropositive participants exhibited symptoms, whereas in other studies asymptomatic carriers among HCW varied from 4.8 to 40% [12,13]. These results indicate that the relatively high percentage of asymptomatic SARS-CoV-2 carriers among HCW may have contributed to the large transmissibility of the disease both in the hospital and in the community. Finally, the last objective of our previous publication - which is also the study subject of the present contribution - was to evaluate demographic and anthropometric characteristics and laboratory parameters measured at the baseline in the cohort as risk factors for infection with SARS-CoV-2 over a time period of six months. In line with previous studies [10,11,14], no specific risk factors could be identified between any of the categorical or continuous variables of the dataset. The only exception was the presence of hypertension at baseline in participants that would remain IgG seronegative, and absence of hypertension in participants that would become seropositive. No additional information was recorded for the hypertensive participants (e.g., the degree of hypertension), but all hypertensive participants were medicated and had blood pressure measurements in the range normal to normal-high, indicating that their hypertensive condition was under control. We concluded that it is possible that participants recognizing themselves as a vulnerable population with comorbidities, such as hypertension, followed safety procedures and social distancing more strictly than participants without known comorbidities, and therefore by adapting their behavior were less likely to become infected [1].

The purpose of the present contribution is to reanalyze the dataset of demographic and anthropometric characteristics and laboratory parameters of the cohort using more advanced statistical methods. Our previous contribution [1] used common univariate statistical analyses, such as the Student t-test for continuous variables and the χ^2^-test for categorical variables, and the conclusions on risk factors for infection with SARS-CoV-2 are counterintuitive because hypertension was defined early on during the pandemic as an important risk factor for adverse health results in the development of the COVID-19 disease. The results of our previous publication were based on a preliminary dataset of n=110 participants that was constructed with the parameters and the evaluation results that were available at that time. Continuous variables included breathing rate (BR), temperature, weight, body mass index (BMI), waist circumference, hip circumference, blood glucose, urea, creatinine, uric acid and cholesterol. Categorical variables included obesity, type-2 diabetes and hypertension. The present contribution uses an updated dataset that includes additional parameters such as heart rate (HR), mean arterial pressure (MAP) and waist-to-hip ratio (WHR), but only considers n=100 participants with complete data. It has been argued before that susceptibility to infection might stem from systemic and physiological factors, rather than solely from local or cellular properties [15]. We hypothesized that a multivariate statistical approach, that considers interactions between different categorical variables or factors, and evaluates the combined effect of multiple continuous variables, better reflects the fact that the human body is a system where physiological variables do not exist in isolation, and therefore might detect differences in susceptibility to infection where univariate analyses do not. For this reason, the aim of the present study is to analyze the dataset of the cohort at baseline using multivariant statistical approaches, such as MANOVA, to identify risk factors for infection with SARS-CoV-2.

**- Describe the confounding factors identified in this study and outline the measures taken to control them.**

*We added a new section at the end of the manuscript to explain the strengths and limitations of our contribution. We identified the factor sex as a confounding factor in our previous publication, which was resolved in the present contribution and led to new results. We do not identify any more confounding factors in the present contribution. See lines 435-447 on page 20 of the manuscript:*

Strengths and limitations. A possible limitation is the relatively small size of the study population. The calculation of the sample size indicated that the complete study population was sufficient for statistical analysis, but the size of the individual subgroups may be too restrictive to adequately reflect the more than seven thousand HCW employed by the HGMEL. A potential strength lies in the considerable homogeneity of our study population, with the vast majority vaccinated against COVID-19 using the same vaccine. Additionally, possible virus infections were identified through two distinct methodologies: qPCR and IgG detection. We detected that an important confounding factor of our previous publication [1] was sex, which was resolved in the present contribution and which led to new results. We do not identify any other confounding factors as we are confident that possible infection and symptoms were correctly detected, without underreporting, and that all demographic, clinical and laboratory parameters were correctly registered.

**- Provide information about the vaccination status of the individuals at the 6-month follow-up and assess the potential protective effects.**

*We added a new paragraph to the section “IgG Seroprevalence Analysis” to explain that most of the study participants were vaccinated, and all were vaccinated with the same vaccine, which contributes to the homogeneity of our study population. The IgG test differentiates between antibodies due to viral infection and the vaccination. The vaccination protects against severe illness, whereas the protection against the infection itself is minimal, short-lived and population dependent. Therefore, we do not consider any potential protective effects. See lines 162-176 on pages 7-8 of the manuscript:*

All participants underwent blood sampling at the beginning of the study (August 2020) and again six months later (January 2021) to determine the seroprevalence of the presence of antibodies against SARS-CoV-2. Specific IgG antibody levels against the SARS-CoV-2 nucleocapsid (N) protein were measured in triplicate by the Enzyme Linked-Immuno Sorbent Assay (ELISA) by a standardized kit from Abcam (Abcam, ab274339, Cambridge, UK), using a microplate reader at 450 nm.

Most participants in this study (85 out of 100, equating to 85%) received their SARS-CoV-2 vaccinations beginning in December 2020, notably after the commencement of the study, and continuing until the follow-up evaluation in January 2021. In all cases, the mRNA-based Pfizer–BioNTech COVID-19 vaccine (brand name Comirnaty) was used. ELISA differentiates between immune responses to infection with SARS-CoV-2 and immune responses caused by all vaccines against COVID-19 that were approved at that time [18]. There is a consensus that vaccination against COVID-19 offers protection against severe illness, whereas the protection against the infection itself is minimal and short-lived [19,20].

**- Discuss the potential implications of this study for the development of long COVID, making reference to these relevant studies (DOI: 10.3390/jcm11020413, DOI: 10.1080/03007995.2022.2081454, DOI: 10.3390/jcm11247314) that highlights a possible association between sex and long COVID.**

*We added an extra paragraph to the Discussion section to discuss the distinct effects of sex on risk to infection with SARS-CoV-2 on the one hand, and the effect of sex on the adverse outcome of COVID-19 disease on the other hand. In the context of COVID-19 disease, we briefly commented on the topic of long COVID, and we included the suggested publications. We prefer not to discuss the topic of long COVID into more detail, because the present contribution is on the initial infection, not on the development of the disease. See lines 384-403 on pages 18-19 of the manuscript:*

The other main finding is that susceptibility to infection with SARS-CoV-2 is modulated by sex. While there were no differences in the physiological state and individual variables between the two groups of female participants (F- and F+), significant differences were observed between the groups of male participants, which were either protected against (M-) or predisposed to (M+) infection. The individual variables that showed significant differences, uric acid, creatinine, waist-to-hip ratio, and mean arterial pressure, were consistently higher in M- compared to M+. All individual continuous variables were within the normal range for M+, but waist-to-hip ratio and mean arterial pressure were above the normal range for M-. These results are counter-intuitive because being overweight and having high blood pressure are known risk factors for the aggravated progression of COVID-19. Therefore, it appears there may be a specific set of risk factors for infection with SARS-CoV-2, and a different set of risk factors for an exacerbated progression of the COVID-19 disease. The present results show that the male sex exhibits specific risk factors for infection with SARS-CoV-2. Other studies have indicated that female COVID-19 patients have an increased risk to develop long COVID [44-46], whereas male patients have higher odds of requiring intensive treatment unit (ITU) admission or of having a fatal outcome [47]. Previously, we have explained the distinct evolution of COVID-19 for both sexes in terms of differences in the structure of their corresponding physiological networks [41,43].

**Reviewer 2**

**In the introduction section, it is stated: "...whereas several participants of the IgG- group were hypertensive." Please indicate the hypertensive grade/severity in those participants suffering from it and its importance in your results.**

*We added some extra explanation with respect to the topic of hypertension in the Introduction section. We do not have additional information on the degree of hypertension, only that all diagnosed hypertensive patients were medicated, and that the blood pressure measurements of all participants were in the normal or normal-high range, such that these do not impact our results. See lines 90-101 on page 4 of the manuscript:*

In line with previous studies [10,11,14], no specific risk factors could be identified between any of the categorical or continuous variables of the dataset. The only exception was the presence of hypertension at baseline in participants that would remain IgG seronegative, and absence of hypertension in participants that would become seropositive. No additional information was recorded for the hypertensive participants (e.g., the degree of hypertension), but all hypertensive participants were medicated and had blood pressure measurements in the range normal to normal-high, indicating that their hypertensive condition was under control. We concluded that it is possible that participants recognizing themselves as a vulnerable population with comorbidities, such as hypertension, followed safety procedures and social distancing more strictly than participants without known comorbidities, and therefore by adapting their behavior were less likely to become infected [1].

**Also, in the introduction, the paragraph beginning with "The results of our previous publication were based on a preliminary dataset of n=110 ..." is somewhat wordy; I suggest reformulating it as follows:
The results of our previous publication were based on a preliminary dataset of n=110 participants that was constructed with the parameters and the evaluation results that were available at that time, including the continuous variables of breathing rate (BR), body temperature, weight, body mass index (BMI), waist circumference, and hip circumference Also, blood glucose, urea, creatinine, uric acid and cholesterol, and the categorical variables of obesity, type 2 diabetes, and hypertension. The present contribution uses an updated dataset that includes additional parameters such as heart rate (HR), mean arterial pressure (MAP), and waist-to-hip ratio (WHR) but only considers n=100 participants with complete data. It has been argued before that susceptibility to infection may be systemic and physiological, apart from a local or cellular property (Caputo et al., 2022).**

*We changed the text in the Introduction section following your suggestion, see lines 109-119 on page 5 of the manuscript:*

The results of our previous publication were based on a preliminary dataset of n=110 participants that was constructed with the parameters and the evaluation results that were available at that time. Continuous variables included breathing rate (BR), temperature, weight, body mass index (BMI), waist circumference, hip circumference, blood glucose, urea, creatinine, uric acid and cholesterol. Categorical variables included obesity, type-2 diabetes and hypertension. The present contribution uses an updated dataset that includes additional parameters such as heart rate (HR), mean arterial pressure (MAP) and waist-to-hip ratio (WHR), but only considers n=100 participants with complete data. It has been argued before that susceptibility to infection might stem from systemic and physiological factors, rather than solely from local or cellular properties [15].

**The discussion is well-written and includes main topics related to leading findings. However, the authors obviate that much research has been done on the Mexican population regarding genetic susceptibility in this topic; a quick search in PubMed can help them in this regard, for example, PMIDs: 35892712, 35395388, 37108839, 35967349, and many others; please consider discussing the lack of assessing the potential genetic factors in your research. A brief paragraph could be essential to add.**

*We added an alternative interpretation of the study results to the Discussion section that discusses genetic susceptibility, and we included the suggested references in the manuscript. We prefer not to discuss the topic of long COVID into more detail, because genetic studies require a separate methodology and study design that is different and complementary to the strategy followed in the present contribution. See lines 419-424 on page 19 of the manuscript:*

An alternative interpretation could be differences in genetic susceptibility between the study groups, where variations in specific alleles can increase susceptibility or resistance, on the one hand to infection with SARS-CoV-2 [50,51] and on the other hand to adverse health outcomes in COVID-19 disease [52-55]. In this context also, the results of the present contribution suggest that genetic susceptibility may be modulated by sex.

**The conclusion section is excessive; the authors should make a concise conclusion according to their results, avoiding being repetitive with the discussion and methods sections.**

*We rewrote and summarized the Conclusion section. See lines 449-455 on page 21 of the manuscript:*

We examined the baseline data of healthcare workers to identify SARS-CoV-2 infection risk factors. We used multivariate statistics, such as MANOVA, to consider physiological factors. We found sex modulates susceptibility to infection. Men have different physiological states affecting their risk. In women, we did not find such distinctions. Men with normal arterial pressure and waist-to-hip ratio resulted to be more at risk. Being overweight and having hypertension worsen the development of COVID-19 disease, but may protect against initial SARS-CoV-2 infection.

**Minor comment: Please homogenize the term SARS-CoV-2. In some places, it is wrong, for example, in the first paragraph of the introduction.**

*We checked the manuscript for the correct spelling of SARS-CoV-2 and hope to have solved all inconsistencies.*
